# A postzygotic de novo* NCDN* mutation identified in a sporadic FTLD patient results in neurochondrin haploinsufficiency and altered FUS granule dynamics

**DOI:** 10.1186/s40478-022-01314-x

**Published:** 2022-02-12

**Authors:** Gaël Nicolas, Myriam Sévigny, François Lecoquierre, Florent Marguet, Andréanne Deschênes, Maria Carment del Pelaez, Sébastien Feuillette, Anaïs Audebrand, Magalie Lecourtois, Stéphane Rousseau, Anne-Claire Richard, Kévin Cassinari, Vincent Deramecourt, Charles Duyckaerts, Anne Boland, Jean-François Deleuze, Vincent Meyer, Jordi Clarimon Echavarria, Ellen Gelpi, Haruhiko Akiyama, Masato Hasegawa, Ito Kawakami, Tsz H. Wong, Jeroen G. J. Van Rooij, John C. Van Swieten, Dominique Campion, Paul A. Dutchak, David Wallon, Flavie Lavoie-Cardinal, Annie Laquerrière, Anne Rovelet-Lecrux, Chantelle F. Sephton

**Affiliations:** 1 Inserm U1245 and CHU Rouen, Department of Genetics and CNR-MAJ, Normandie University, UNIROUEN, F-76000 Rouen, France; 2 Department of Psychiatry and Neuroscience, Laval University, Quebec City, QC Canada 2325, rue de l’Université, G1V 0A6; 3 CERVO Brain Research Centre, Laval University, Quebec City, QC Canada 2601, chemin de la Canardière, G1J 2G3; 4 Inserm U1245 and CHU Rouen, Department of Pathology, Normandie University, UNIROUEN, F-76000 Rouen, France; 5 Institut Intelligence et Données, Laval University, Quebec City, QC Canada; 6 Department of Biochemistry, Microbiology and Bioinformatics, Laval University, Quebec City, QC Canada; 7 Lille Neuroscience and Cognition (Inserm UMRS1172) Alzheimer and Tauopathies, Laboratory of Excellence Distalz (Development of Innovative Strategies for a Transdisciplinary Approach to ALZheimer’s Disease), University of Lille, CHU Lille, Lille, France; 8 Department of Neuropathology, University of Lille, CHU Lille, Lille, France; 9 INSERM, CNRS U1127, Institut du Cerveau, Sorbonne Université, ICM, Paris, France; 10 Laboratoire de Neuropathologie R. Escourolle, AP-HP, Hôpital de la Pitié-Salpêtrière, Paris, France; 11 CEA, Centre National de Recherche en Génomique Humaine, Université Paris-Saclay, 91057 Evry, France; 12 Memory Unit, Department of Neurology, Hospital de la Santa Creu i Sant Pau, Biomedical Research Institute Sant Pau, Universitat Autònoma de Barcelona, Barcelona, Spain; 13 Centro de Investigación Biomédica en Red Enfermedades Neurodegenerativas (CIBERNED), Madrid, Spain; 14 Neurological Tissue Bank of the Biobank-Hospital Clinic-IDIBAPS, Barcelona, Spain; 15 Division of Neuropathology, Department of Neurology, Medical University of Vienna, Vienna, Austria; 16 Dementia Research Project, Department of Brain and Neurosciences, Tokyo Metropolitan Institute of Medical Science, Setagaya City, Japan; 17 Department of Neurology and Alzheimer Center, Erasmus Medical Center, Rotterdam, The Netherlands; 18 Department of Internal Medicine, Erasmus Medical Center, Rotterdam, The Netherlands; 19 UNIROUEN, Inserm U1245 and CHU Rouen, Department of Neurology and CNR-MAJ, Normandie University, F-76000 Rouen, France

**Keywords:** FUS (fused in sarcoma), Frontotemporal dementia (FTD), NCDN (neurochondrin), Norbin, Genetic variant, De novo mutation, FTLD-FET, Neurodegeneration, mGluR1/5, Cytoplasmic granules

## Abstract

Frontotemporal dementia (FTD) is a heterogeneous clinical disorder characterized by progressive abnormalities in behavior, executive functions, personality, language and/or motricity. A neuropathological subtype of FTD, frontotemporal lobar degeneration (FTLD)-FET, is characterized by protein aggregates consisting of the RNA-binding protein fused in sarcoma (FUS). The cause of FTLD-FET is not well understood and there is a lack of genetic evidence to aid in the investigation of mechanisms of the disease. The goal of this study was to identify genetic variants contributing to FTLD-FET and to investigate their effects on FUS pathology. We performed whole-exome sequencing on a 50-year-old FTLD patient with ubiquitin and FUS-positive neuronal inclusions and unaffected parents, and identified a de novo postzygotic nonsense variant in the *NCDN* gene encoding Neurochondrin (NCDN), NM_014284.3:c.1206G > A, p.(Trp402*). The variant was associated with a ~ 31% reduction in full-length protein levels in the patient’s brain, suggesting that this mutation leads to NCDN haploinsufficiency. We examined the effects of NCDN haploinsufficiency on FUS and found that depleting primary cortical neurons of NCDN causes a reduction in the total number of FUS-positive cytoplasmic granules. Moreover, we found that these granules were significantly larger and more highly enriched with FUS. We then examined the effects of a loss of FUS function on NCDN in neurons and found that depleting cells of FUS leads to a decrease in NCDN protein and mRNA levels. Our study identifies the NCDN protein as a likely contributor of FTLD-FET pathophysiology. Moreover, we provide evidence for a negative feedback loop of toxicity between NCDN and FUS, where loss of NCDN alters FUS cytoplasmic dynamics, which in turn has an impact on NCDN expression.

**Supplementary Information:**

The online version contains supplementary material available at 10.1186/s40478-022-01314-x.

## Introduction

Frontotemporal dementia (FTD) is a common form of dementia characterized by a progressive neuronal loss, primarily across the frontal and temporal lobes, leading to changes in executive functions, personality, abnormal behaviors and language impairments [43, 54]. FTD is a heterogeneous clinical, genetic and pathological disorder and is broadly referred to as frontotemporal lobar degeneration (FTLD). Subtypes of FTLD are further categorized based on the composition of major proteins found in insoluble cellular inclusions. The three main subtypes of FTLD are defined by the protein composition of these inclusions: FTLD-Tau, FTLD-TDP (TAR-DNA binding protein-43), and FTLD-FET [51]. FTLD-TDP and FTLD-FET are both characterized by Tau-negative, ubiquitin-positive deposits and differ by the actual proteins involved in these deposits. FET refers to the family of proteins composed of FUS (Fused in Sarcoma), EWS (Ewing Sarcoma Breakpoint region 1), and TAF15 (TATA-binding protein-associated factor 15) proteins, which are the main components of the aggregates [74]. FTLD-FET accounts for 5–10% of all FTLD cases and it is further subdivided into three subtypes: atypical-FTLD with ubiquitin-positive inclusions (aFTLD-U), basophilic inclusion body disease (BIBD), and neuronal intermediate filament inclusion body disease (NIFID) (for review, see reference [58]).

Loss of function or missense variants in the gene encoding FUS, one of the main components of the aggregates of FTLD-FET cases, typically cause amyotrophic lateral sclerosis (ALS) [44, 82, 91], a motor neuron disease that shares overlapping genetic and pathological features with FTD [3]. Such ALS mutations are inherited or occur de novo [60] and post-mortem analysis of patient tissues show nuclear depletion and cytoplasmic aggregation of FUS [35, 78], similar to that of FTLD-FET [31, 51, 66]. However, very few *FUS* mutations have been identified in FTLD-FET patients or even in cases of ALS-FTLD [31, 81].

FUS is a ubiquitously expressed DNA/RNA binding protein that is predominantly localized to the nucleus of cells [11, 74]. It is capable of nucleocytoplasmic shuttling via its C-terminal proline-tyrosine nuclear localization sequence (PY-NLS) and nuclear export sequence (NES) [16, 41, 93]. In the nucleus, FUS regulates transcription, [4, 13, 75, 89] splicing [14, 32, 45] and DNA damage repair [42]. In the cytoplasm, FUS regulates mRNA transport and stability, miRNA processing and translation regulation through its interactions with RNA [20, 62, 69, 79]. FUS binds several thousand RNAs at coding, non-coding and 5’- and 3’-UTR regions [27, 32, 46, 55, 63], mediated through its RNA recognition motif (RRM), zinc finger (ZnF) domain and three arginine-glycine-glycine (RGG) boxes [49]. At its N-terminal, FUS contains a low complexity domain (LCD) that contributes to its liquid–liquid phase separation (LLPS) properties as well as its interactions with RNA and other proteins [17]

In this study, we identified a postzygotic de novo mutation in the *NCDN* gene (also known as *Norbin),* which was predicted to result in haploinsufficiency in a patient with sporadic FTLD-FET. Our study investigates its contribution to FTLD-FET and the effects on FUS pathology. We show that depleting primary cortical neurons of NCDN leads to significant changes in FUS solubility and association with cytoplasmic granules. Moreover, the depletion of FUS from cells leads to a decrease in NCDN levels. Together, our data suggest that there is a negative feedback loop of toxicity between NCDN and FUS, where loss of NCDN alters FUS cytoplasmic dynamics and loss-of-function or aggregation of FUS could promote neuronal dysfunction through the misregulation of NCDN expression.

## Materials and methods

### Source of materials

Reagents were obtained from the following sources: PhosSTOP (4906845001), cOmplete EDTA-free Protease Inhibitor Cocktail (11836170001), MISSION® shRNA: TRCN0000225722 (FUS-KD-1), TRCN0000225724 (FUS-KD-2), TRCN0000010598 (hFUS-KD-1), TRCN0000119421 (NCDN-KD-1), TRCN0000119417 (NCDN-KD-2) and SHC002 (pLKO.1-puro Non-Mammalian shRNA Control Plasmid) are from Sigma-Aldrich; Cycloheximide (CHX) (AC357420010) from Thermo Fisher Scientific; PDL- and Laminin-PDL coated coverslips from Neuvitro Corporation (GG-12-PDL and GG-12-LAMININ). The following antibodies were used for immunofluorescence experiments: antibodies to FUS/TLS (HPA008784) and MAP2 (MAB3418) from Sigma-Aldrich; goat anti-rabbit Alexa Fluor® 488 (A-11034) and goat anti-Mouse Alexa Fluor® 546 (A-11030) from Thermo Fisher Scientific. The following antibodies were used for western blotting experiments: FUS/TLS (A300-302A) from Bethyl Laboratories;and FUS/TLS (sc-47711) from Santa-Cruz; NCDN (gift from Dr Flajolet), β-actin (2066) and GAPDH (G9545) from Sigma. Secondary antibodies: IRDye® 680RD Goat anti-Mouse IgG (926-68070), IRDye® 800CW Goat anti-Rabbit IgG (926-32211) from LI-COR Biosciences. Immunohistochemical studies were carried out using antibodies directed against α-synuclein (diluted 1/75, Eurobio, les Ulis, France), the PHF tau (AT8, 1/20, Innogenetics, Gent, Belgium), ubiquitin (1/100, Agilent, Les Ulys, France), α-internexin (1/75; Life technologies- Invitrogen, Courtaboeuf, Villebon-sur-Yvette, France), TDP43 (1/1000; Proteintech Europ, Manchester, UK) and FUS (1/100; Euromedex, Souffelweyersheim, France).

### Neuropathological examination and immunohistochemistry

An autopsy restricted to the brain and cervical spinal cord was performed. After extraction of the brain which weighed 1080 g, and after excluding gross macroscopical asymmetries, 1 cm-thick coronal slices obtained from the left hemisphere as well as a cerebellar sample were stored at −70 °C until use. The right hemisphere as well as the whole brainstem and cerebellum were fixed in a 10% formaldehyde solution buffer. Tissue samples were taken from multiple areas including anterior upper and middle frontal gyrus, superior temporal gyrus, temporal pole, inferior parietal gyrus, anterior cingular gyrus, insular and motor cortex, calcarine fissure, hippocampus, nucleus basalis of Meynert, amygdala, basal ganglia, cerebral peduncles, pons, medulla oblongata and cerebellum (vermis, right hemisphere and dentate nucleus). Seven-micrometer sections were cut from paraffin-embedded blocks and stained with haematoxylin–eosin. Immunohistochemical studies were carried out using antibodies directed against α-synuclein (diluted 1/75, Eurobio, les Ulis, France), the PHF tau (AT8, 1/20, Innogenetics, Gent, Belgium), ubiquitin (1/100, Agilent, Les Ulys, France), α-internexin (1/75; Life technologies- Invitrogen, Courtaboeuf, Villebon-sur-Yvette, France), TDP43 (1/1000; Proteintech Europ, Manchester, UK), FUS (1/100; Euromedex, Souffelweyersheim, France), and TAF15 (1/200; Ozyme, St Cyr l’école, France). Immunohistochemical procedures included a microwave pre-treatment protocol to aid antigen retrieval (pre-treatment CC1 kit, Ventana Medical Systems Inc, Tucson AZ). Incubations were performed for 20, 32 or 60 min at room temperature using the Ventana Benchmark XT system. After incubation, slides were processed by means of the Ultraview Universal DAB detection kit (Ventana).

### Protein extraction and western blotting from brain

Sequential extraction of proteins from frontal cortex was performed as described in [57]. Briefly, frozen brain samples were extracted at 100 mg brain samples in 1 mL volume buffer, each extraction step being followed by a centrifugation step et 120 000 g, 30 min, 4 °C. Extraction buffer all contained a protease inhibitor cocktail (P8340, Sigma, Saint-Louis, MO, USA) and included (i) high-salt (HS) buffer containing 50 mM Tris–HCl, 750 mM NaCl, 10 mM NaF, 5 mM EDTA, pH 7.4, (ii) HS buffer containing 1% Triton-X (HS-TX), (iii) RIPA Lysis and extraction buffer (from ThermoFisher Scientific, Waltham, MA USA), (iv) 2% sodium dodecyl sulphate buffer (2% SDS). At each extraction step pellets were washed with the corresponding buffer to prevent carry over. Finally, the 2% SDS insoluble pellet was extracted in 70% formic acid (Ac. Form) and evaporated in a SpeedVac system. The dried pellet was resuspended in 3X Laemmli buffer containing 100 mM DTT. For immunoblot analysis, equivalent amounts of each fraction were resolved by 10% sodium dodecyl sulphate–polyacrylamide gel electrophoresis and transferred to nitrocellulose membranes (Bio-Rad Laboratories, Hercules, CA, USA). Membranes were blocked with phosphate-buffered saline containing 0.05% Tween and 5% non-fat dried milk and probed with anti-FUS antibody (1/5000; Bethyl Laboratories, Inc. Montgomery, TX, USA). Gel loading was normalized by Stain-Free detection of total proteins using a Geldoc™ EZ Imager (BioRad laboratories). The Stain-Free signal obtained in each lane was quantified with the ImageLab™ software (Bio-Rad Laboratories). Primary antibody was detected with horseradish peroxidase-conjugated anti-rabbit (Jackson ImmunoResearch, West Grove, PA, USA). Signals were detected with chemiluminescence reagents (ECL Clarity, Bio-Rad Laboratories) and acquired with a GBOX (Syngene, Cambridge, UK), monitored by the Gene Snap software (Syngene). The signal intensity was quantified using the Genetools software (Syngene).

### Exome sequencing and genetic analyses

The legal guardian of the patient and both parents provided written consent for genetic analyses in a research setting (RBM-0259; this study was approved by the Ile de France II ethics committee). DNA was isolated from whole blood of the patient and both unaffected parents using standard procedures. Exomes were sequenced using the Illumina technology following capture using an Agilent Human all exons capture kit, V4UTR, by the Integragen society (Evry, France) with an average depth of coverage of 100x. Parenthood was checked prior to WES analysis using informative microsatellite analysis in all three members of the trio and was verified in exome sequencing data. Reads were mapped to the 1000 Genomes GRCh37 build using BWA 0.7.5a.10. Picard Tools 1.101 was used to flag duplicate reads. We applied GATK for indel realignment, base quality score recalibration and single nucleotide polymorphisms, and indels discovery using the Haplotype Caller across all samples simultaneously according to GATK 3.3 Best Practices recommendations. Variants were annotated using a homemade pipeline using SnpEff, SnpSift and numerous data sources including clinVar, OMIM, DenovoDB and GTEX tissular expression and gnomAD variants frequency. In order to identify de novo candidate variants, we selected high confidence variants in the proband (genotype quality ≥ 90 and read depth ≥ 10), then subtracted all variants detected in at least one parent.

Rare (allele frequency in gnomAD < 1%) variants in the following genes were interpreted and no putatively pathogenic variant was found: *MAPT*, *GRN*, *VCP*, *FUS*, *TARDBP*, *CHMP2B*, *SQSTM1*, *OPTN*, *CHCHD10*, *HNRNPA*, *HNRNPA2B1*, *UBQLN2*, *TBK1*.

For targeted Sanger sequencing and Snapshot analyses, the proband’s DNA was isolated from blood using the Flexigen kit (Qiagen), and from frozen brain samples using the DNA Blood and Tissue kit (Qiagen). Brain regions included frontal cortex, hippocampus, occipital cortex, parietal cortex and temporal cortex. Snapshot analyses were applied to DNA isolated from blood and from all the above-mentioned brain regions. The presence of the G > A mutation was analysed using the SNaPshot (PE Applied Biosystems) technique, according to the manufacturer’s instructions. Briefly, blood and brain-extracted DNA were PCR amplified using primers flanking the mutation (Forward primer: 5’-TGGTCCTGCTCCATCTCAAG-3’; Reverse primer: 5’-TAGAGGGTCTTGGCATAGCG-3’). The PCR product was then submitted to primer extension with fluorescent ddNTPs and the following primer: 5’-GTGCGGATCCTGGGTGCCTG-3’. The extended primer was finally submitted to electrophoresis on an automated sequencer (ABI 3500; Applied Biosystems), and fluorescence was analyzed using the GeneMapper software (Applied Biosystems).

### Replication analysis

We gathered exome sequencing data or DNA samples from multiple international cases with FTLD-FET (Additional file 1: table 1) [40]. All patients or legal guardians provided written consent for genetic analyses.

Overall, we performed WES (i) from DNA isolated from blood of two patients and from brain of 4 patients from France and (ii) from DNA isolated from brain of 5 patients from Spain. One WES was sequenced using the same procedures as the patient carrying the *NCDN* nonsense variant. The other patients were also sequenced using the Illumina technology following capture using an Agilent Human all exons capture kit, V5UTR, at the CNRGH (CEA, Evry, France) with an average depth of coverage of 130x. Bioinformatics pipelines applied to all WES were the same as described above with the exception of sequencing of parents, whose DNA was not available.

In addition, we performed Sanger sequencing of the *NCDN* gene in three patients from Spain, two previously reported Japanese patients [40] and in four patients from France, then we analyzed the *NCDN* gene sequencing data out of WES data previously generated in 5 Dutch patients. The last 5 patients underwent exome sequencing using a SeqCap V2 capture kit and Illumina sequencing with an average depth of coverage of 60x.

### Primary neuron culture

Dissociated rat or mouse cortical neurons were prepared from neonatal pup brains as described previously [56]. Cells were plated at a density of 62 cells/mm^2^ on poly-D-lysine (PDL)-coated culture plates or at a density of 50 cells/mm^2^ on glass coverslips laminin-coated on PDL layer (Neuvitro Corporation). Neurobasal media supplemented with serum-free B-27™ (50:1; Gibco, 17504001), penicillin/streptomycin (50U/mL; 50 μg/mL; Gibco, 15140148) and 0.5 mM L-GlutaMAX (Invitrogen, 35050061) was used as growth medium. When plating the cells, fetal bovine serum (5%; Hyclone SH30071.03) was added. At DIV5, half of the culture medium was removed and replaced by serum-free growth medium containing Ara-C (5 μM; Sigma, C1768) to limit proliferation of non-neuronal cells. Neurons were then fed twice a week by replacing half of the culture medium with fresh serum- and Ara-C-free growth medium.

### Cell culture

Neuro-2a and HEK293T cell were cultured in complete medium: 10% fetal bovine serum (Gibco, 12483020) and DMEM (Gibco, 11965-092) and grown under standard culture conditions (37 °C, 5% CO2, 95% air).

### Lentivirus production and infection

For lentivirus production, HEK293T cells were cultured in complete medium and grown to 70% confluence, followed by co-transfection with lentivirus packaging vectors (VSVG and Δ8.9) and pLKO.1-puro vectors (CTL, FUS-KD1, FUS-KD2, hFUS-KD1, NCDN-KD-1 and NCDN-KD-2) using FUGENE6 (Promega, E2691) following the manufacturer’s instructions. 24 h post-transfection, the medium was replaced by neuron growth medium and 48 h post-transfection, the condition medium was filtered through a 0.45 μm filter, snap frozen in liquid nitrogen and stored at -80 °C until use. Lentivirus titer was obtained using the NucleoSpin® RNA Virus prep kit (Takara Bio, 740956.10) and Lenti-X™ qRT-PCR Titration Kit (Takara Bio, 631235). Upon titer determination, approximately 2–4 × 10^8^ viral copies/mL were added to DIV8-9 primary cortical neurons.

### Immunofluorescence staining of neurons

Immunofluorescence of primary cortical neurons were performed as previously described [67, 69]. Cells were grown on PDL-coated coverslips and fixed with 4% paraformaldehyde (PFA) solution (4% sucrose, 100 mM phosphate buffer, 2 mM NaEGTA pH7.4) for 10 min at room temperature (RT). Samples were washed three times for 5 min with phosphate buffer saline (PBS) and then incubated during 30 min in blocking/permeablization solution (0.1% Triton X-100 and 2% normal goat serum (NGS)) at RT. Samples were incubated in primary antibody at 4°C, overnight. Samples were washed three times 10 min with 1X PBS and incubated in Alexa Fluor® 488 secondary antibodies diluted in blocking/permeabilization solution for 1 h, RT. Coverslips were washed three times for 10 min with 1X PBS and then mounted with ProLong™ Gold Antifade mounting media containing DAPI (Thermo Fisher, P36935).

### Confocal microscopy image acquisition

Neurons were imaged using a ZEISS LSM700 inverted confocal microscope using constant acquisition settings within image groups from each type of immunofluorescence measurement. Images were acquired using a 63 × oil immersion objective (1.4 NA, Zeiss), in z-stack mode with an interval of 0.45 μm and analysed by ZEN imaging software (Zeiss) to generate maximal intensity projections (MIP) or 3D images. Confocal images showing the cytosolic localization of FUS were captured using settings where the nuclear FUS signal was saturated (Fig. 5). Whereas the confocal images showing the depletion of FUS by shRNA-FUS lentiviruses were captured using settings that did not contain saturated pixels for FUS (Fig. 7).

### Quantitative image analysis

*Quantification of cytoplasmic NCDN signal:* Using a triangle threshold on the MIP of the DAPI signal, we created a segmentation mask of the nuclei. Objects with areas smaller than 20 pixels were removed. Binary hole filling and dilation (kernel size: 3) was performed on the remaining objects to generate the nuclei segmentation map. The foreground of the NCDN channel signal was extracted in each image slice by 3D Gaussian smoothing of all the frames in the z-stack with a sigma of 0.5 followed by the application of a triangle threshold and removal of objects smaller than 30 pixels. For the analysis of NCDN intensity, the nucleus segmentation map was subtracted from the foreground mask to obtain the segmentation mask of cytosolic NCDN. For each experimental week, the fluorescence intensity in each image was normalized by the median cytosolic NCDN intensity of all neurons treated with the non-targeted scramble. Outliers greater than 2 standard deviations from the mean of each condition were removed. To estimate the median distribution with a 95% confidence interval (CI) we resampled the data points with replacement 10 000 times.

*Quantification of FUS granules fluorescence intensity:* For the segmentation of FUS granules in the confocal z-stacks, we used the 3D spot filter of the Allen Cell Structure Segmenter (Chen et al*.,* 2020 https://doi.org/10.1101/491035). We used auto contrast pre-processing and 3D gaussian smoothing (σ = 1). The segmentation parameters were adjusted for each experimental week. (range: 0.75–1,0.015–0.045). Thresholds were applied to remove detections of non-specific staining or measurement artifacts from the segmented clusters (area under 5 pixels or over 300 pixels, Intensity above 30,000 photon counts). The maximum intensity of each cluster was computed to generate the intensity distribution histogram of the FUS granules. The histogram was fitted using a skew normal function and the peak of the fitted distribution was calculated. For each experimental week, the peak position of the fitted distribution of all the images of the CTL condition were used for normalization. Outliers greater than 2 standard deviations from the mean of each condition were removed. To estimate distribution with a 95% confidence interval (CI) we bootstrapped the data points [5]. For the bootstrap analysis the data points were resampled with replacement 10,000 times and their bootstrapped differences were plotted.

*Quantification of FUS granules volume, number and area:* Confocal images of primary neurons were used for 3D analysis using Imaris software. A region of interest (ROI) was defined for all cell bodies of each data set. For each ROI, FUS signal was detected using 488 nm channel using the Imaris spot detection module. An arbitrary mean intensity threshold was used to detect the number of granule spots, defining a minimum detectable size of 0.01um and subtracting the detection coming from the cell nucleus. A minimum number of 16 cells per group were analysed. The volume, number and area of FUS-positive granules were calculated and represented as mean per cell analysed. An Ordinary One-way ANOVA with multiple comparisons using Turkey test was used to compare the different groups for the statistical analysis.

### Western blotting and subcellular fractionation

Cortical neurons (DIV16) were washed once with ice cold 1X PBS pH7.4 (Gibco, 10010–023) and then lysed in radioimmunoprecipitation assay buffer (RIPA) buffer (20 mM Tris–HCL pH 8.0, 1 mM NaEDTA pH8.0, 0.5 mM NaEGTA pH 8.0, 1% Triton X-100, 150 mM NaCL, 1X protease inhibitors EDTA-free, 1X PhosSTOP (Roche)) to obtain total cell lysates (TCL). Neuro-2a cells were lysed in polyribosome lysis buffer (PLB) (20 mM Tris–HCL pH7.4, 5 mM MgCl2, 100 mM KCl, 1% NP-40, 1 mM DTT, 20U/µl SUPERase Inhibitor, 1X protease inhibitors EDTA-free, 1X PhosSTOP (Roche)), then subcellular fractionation of lysates was performed by centrifugation (10,000 × g, 10 min, 4 °C) to obtain supernatant (S1) and pellet (P1) fractions. The P1 fraction was then resuspended in RIPA buffer. All protein extracts were prepared in 1X Laemmli buffer and boiled (5 min, 95 °C) for western blot analysis. Samples were resolved on SDS–polyacrylamide gels and transferred to nitrocellulose membranes followed by standard western blotting procedure as described in [69]. Membranes were imaged using the LI-COR Odyssey imaging system. Analysis of signal intensity was done using Image Studio Lite Software Version 5.2. Quantification of FUS expression in the subcellular fractions was normalized to GAPDH for TCL and S1 and ponceau red for P1.

### Quantitative RT-PCR

Total RNA from cells were extracted using TRIzol™ Reagent (Invitrogen) following the manufacturer protocol. RNA extracts were treated with DNase I (Roche, 4716728001), and cDNA was synthesized using High-Capacity cDNA Reverse Transcription Kit (Applied Biosystems, 4368813). Quantitative RT-PCR reactions of 10 μL contained 25 ng cDNA, 150 nM of each primer and 5 µL PowerUp™ SYBR™ Green Master Mix (Applied Biosystem, A25741). All reactions were performed in triplicate on a QuantStudio 5 Real-Time PCR System (Applied Biosystem) and relative mRNA levels were calculated by the comparative threshold cycle method using U36B as the internal control.

The following primer pairs were used:

U36B (Forward 5’- CGTCCTCGTTGGAGTGACA-3’; Reverse 5’- CGGTGCGTCAGGGATTG -3’),

TDP-43 (Forward 5’-AACTGAGCAGGATCTGAAAGACTATTT-3’; Reverse 5’-CCCTTTCGAGTGACCAGTTTTAA-3’),

FUS (Forward 5’-CAGCTATCGACTGGTTTGATG-3’; Reverse 5’-CGATTGAAGTCAGCTCGGCG-3’).

NCDN (Forward 5’-GCGCCATCGTGAAGTGTGA-3’; Reverse 5’-CCCGCCTTGCCCAACTGT-3’).

### Statistical analyses

At least n = 3 biological experiments were performed for every statistical analysis using Microsoft Excel 2013; this means independent cell cultures were performed for each biological experiment. A Student’s t-test at 95% confidence was used for the comparison of two groups. Statistical analysis was performed for fractionation experiments comparing the relative protein intensities and for QPCR. Each statistical analysis and the number of biological experiments are indicated in the figure legends. All statistical analysis giving *p* < 0.05 are significant.

## Results

### Identification of a de novo variant in the *NCDN* gene in a FTLD-FET patient

We aimed to identify a genetic determinant of FTLD-FET in a female patient (AH-11-02) with early-onset, sporadic behavioral variant of FTD (bvFTD). The patient had no personal history of psychiatric or neurological diseases including a normal development except for a history of dyslexia. In her early forties, she started to show behavioral troubles with a progressive psychomotor slowing, impairment of interpersonal relationships, mistakes at work, and loss of self-care. By the age of 44, she experienced a sharp decline in her performance at work and severe interpersonal relationship issues led to termination of her employment. Shortly thereafter, she was hospitalized at the age of 45 years and displayed apragmatism, loss of social adaptation, lack of motivation and aggressive behavior. Treatment with the antidepressant citalopram or antipsychotic drugs aripiprazole and olanzapine or sismotherapy, did not lead to any improvement in behavior. A CT scan performed at age 47 showed fronto-temporal atrophy (data not shown). Neurological and psychological assessment showed motor and verbal initiation impairment gathering apathy, reduced spontaneous speech, and loss of emotional reactivity. However, she occasionally presented with episodes of extreme impulsivity, aberrant motor behavior and unmotivated laughter. Signs of delusion or mood disorders were never reported. Gradually, the cognitive and behavioral syndrome increased leading to severe perseverations, hyperorality and a complete loss of language. The patient died at the age of 50 and an autopsy restricted to the brain was performed. She had no family history of any neurodegenerative or psychiatric disease, except late onset dementia in the maternal grandfather who died at age 96. Both parents were unrelated and were healthy at age 80 and 75, respectively for the father and the mother, at the time of death of their daughter.

**Fig. 1:**
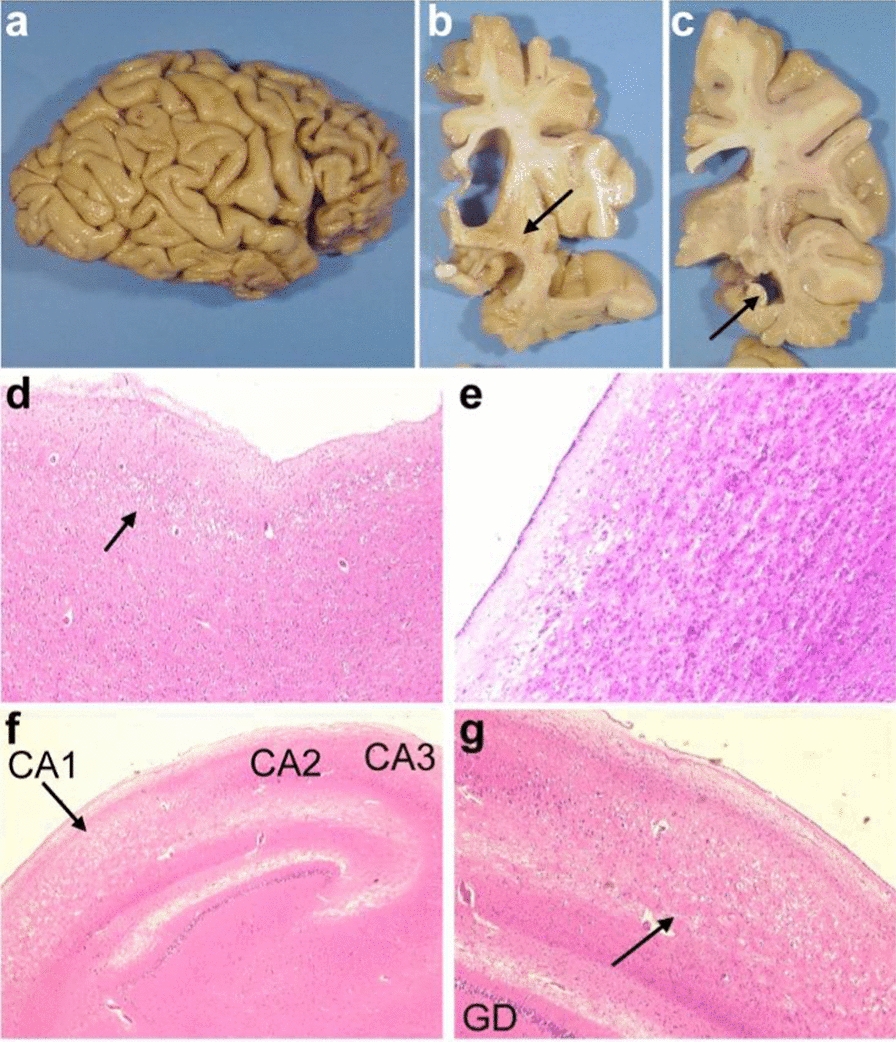
Representative macroscopic and microscopic findings from the FTLD patient’s brain**. a** External view of the right hemisphere showing frontal and temporal atrophy with sparing of the cingular cortex along with the parietal and occipital lobes. **b–c** Coronal sections passing through the atrophic caudate nucleus, putamen and pallidum (arrow, **b**) and atrophic hippocampus (arrow, **c**). **d–g** Histological examination [H&E, OM × 20] showing absent lamination of the anterior frontal cortex with vacuolization of layers II and III (arrow, **d**), severe gliosis in the caudate nucleus (**e**) and neuronal depletion replaced by gliosis of the CA1 field of the Ammon’s horn (arrows, **f** and **g**; H&E, OM × 20; OM × 100 respectively). H&E: haematoxylin and eosin stains; OM: original magnification

Post-mortem examination of the brain revealed severe atrophy in the frontal and temporal lobes (Fig. 1a). The substantia nigra and the locus coeruleus were moderately depigmented. On coronal sections of the right hemisphere, the cerebral white matter was irregularly discoloured. The caudate nucleus, putamen and pallidum were atrophic (Fig. 1b), as well as the hippocampus (Fig. 1c). Ventricular dilatation was also noted, more pronounced in the anterior horns. The cerebellum and brainstem displayed no macroscopical lesions (data not shown).

Histological examination revealed neuronal loss in the pigmented nuclei of the brainstem, particularly the substantia nigra pars compacta, associated with interstitial pigment deposition and reactive gliosis, but Lewy bodies were never observed, even by means of α-synuclein immunohistochemistry (data not shown). In the cerebellum, neither spongiosis nor internal granular cell layer atrophy were noted. Purkinje cells were preserved. No spinal cord lesions were noted. Frontal and temporal cortices except for the superior and middle temporal gyri were severely affected (Fig. 1d). The lesions associated severe neuronal loss, microvacuolization of layers II and III, as well as vanishing cortical lamination. The rare residual pyramidal neurons of layer III contained lipofuscin accumulation. Severe neuronal loss was also observed in the basal ganglia, with almost no neurons and severe gliosis of the caudate nucleus (Fig. 1e). In the hippocampus, the dentate gyrus, the CA4, CA3 and CA2 fields of the Ammon’s horn were preserved, whereas the CA1 field, presubiculum and subiculum were almost devoid of neurons and were replaced by gliosis, consistent with hippocampal sclerosis (Fig. 1f, g). The parietal and cingular cortices had a normal lamination with mild neuronal loss and the occipital cortex was spared.

**Fig. 2:**
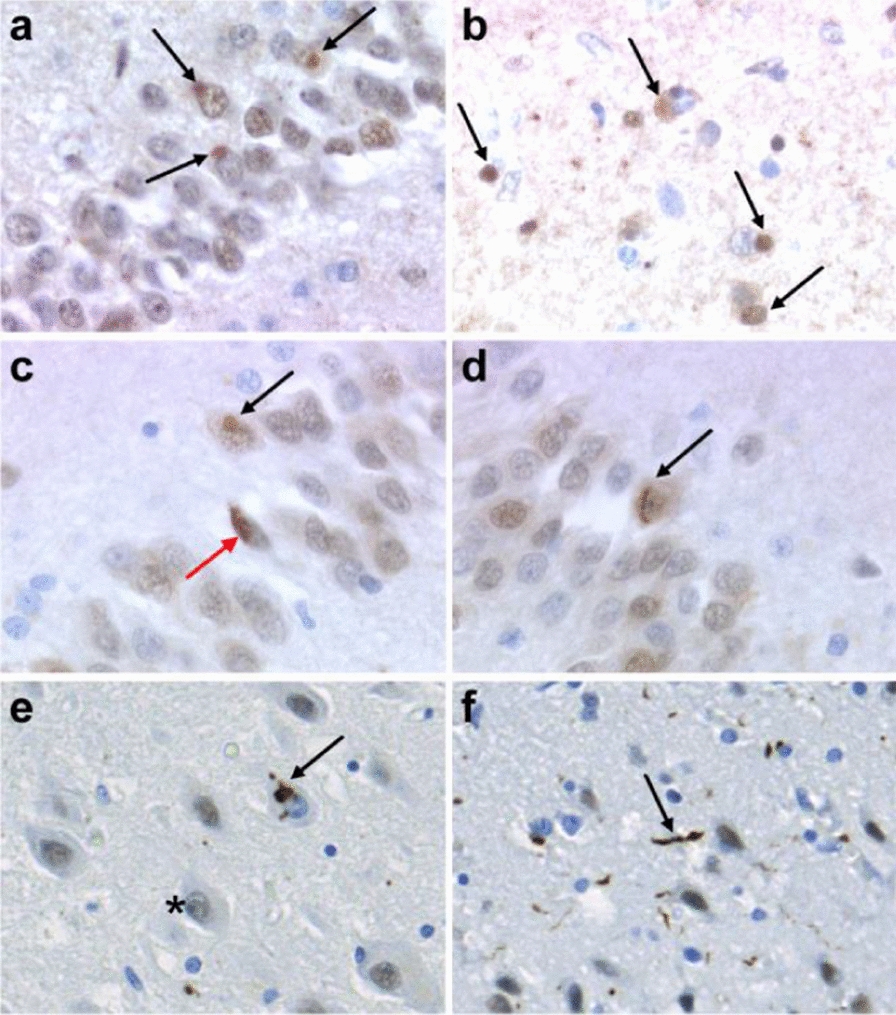
Immunostaining of brain tissue from the FTLD-FET patient. **a–d** Immunohistochemical lesions in the hippocampus and frontal cortex. Ubiquitin immunolabeling displays rounded intra-cytoplasmic inclusions within the dentate gyrus (black arrows, **a**) but with no deformation of the nucleus [OM × 400]. Similar intra-cytoplasmic inclusions were observed in the anterior frontal cortex using FUS immunohistochemistry (black arrows, **b**), associated to FUS-positive either rounded (black arrow, **c**) or elongated granular intracytoplasmic inclusions (red arrow, **c**) [OM × 400] or filamentous curvilinear intranuclear inclusions in the dentate gyrus (black arrow, **d**) [OM × 400]. **e** TAF15 immunostaining displaying normal, finely granular nuclear staining (asterisk) contrasting with loss of TAF15 nuclear immunostaining resulting in cytoplasmic aggregates (black arrows) with **f**, intracortical neuritic accumulations (black arrow). OM: original magnification

Pathological protein aggregates were observed in the affected brain regions. Ubiquitin-positive, round-shaped inclusions were observed in the neuronal perikarya of the dentate gyrus, anterior frontal cortex and temporal pole (Fig. 2a and b). These inclusions were negative for Tau and TDP-43 (data not shown). However, intra-cytoplasmic and intranuclear FUS-positive neuronal inclusions were observed in the dentate gyrus and in the frontal cortex (Fig. 2c and d). Intra-cytoplasmic round-shaped neuronal inclusions were concentrated in the vicinity of the nuclei. They were relatively homogeneous whereas intranuclear inclusions had a granular or vesicular pattern, some of them with filamentous or curvilinear appearance (Fig. 2c and d). Moreover, some glial inclusions were observed in the white matter. These inclusions were immunolabelled by the anti-TAF15 antibody (Fig. 2e and f). These findings led to a final diagnosis of FTLD-FET, atypical FTLD-U subtype according to Neumann and Mackenzie [58].

**Fig. 3:**
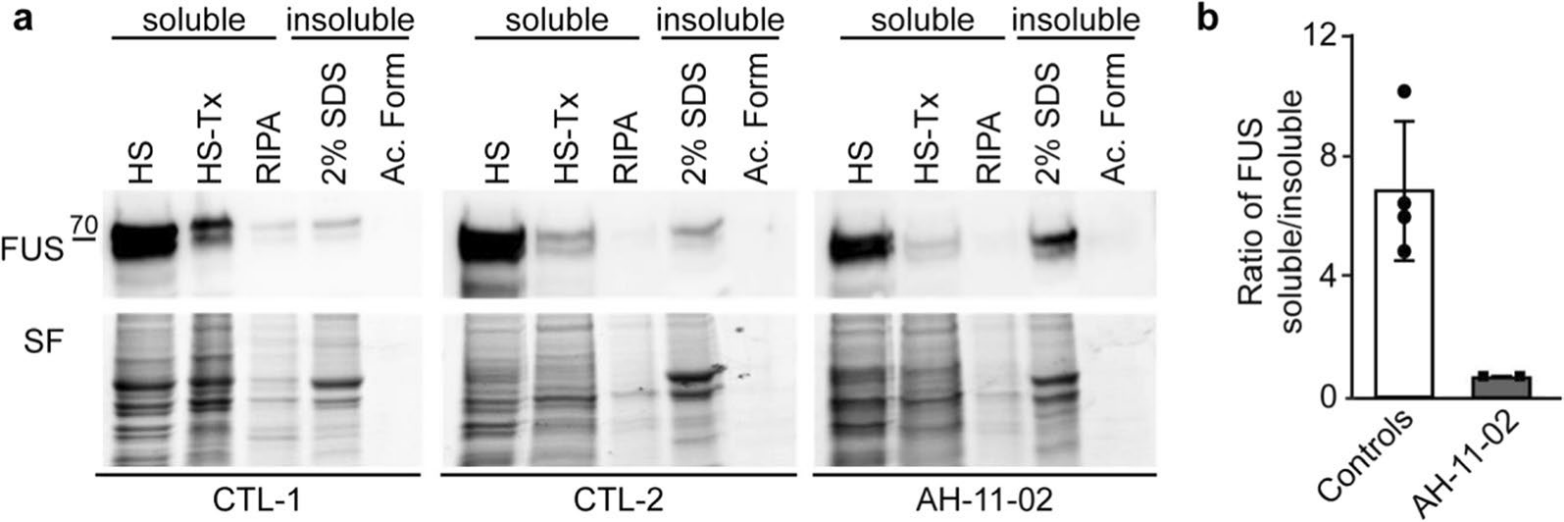
Increased insolubility of FUS in the FTLD patient’s brain compared to controls **a** Western blot analysis of proteins extracted from frozen brain tissue, from controls (CTL-1, -2) or affected patient (AH-11–02). FUS staining was performed on samples resulting from sequential brain extraction, using buffers with increasing solubilisation properties. Total protein was used as loading control with Stain-Free technology (SF). Three independent extractions per individual were performed. Representative blots and Stain-Free staining are shown. **b** Graph representing the ratio of FUS bands intensity in the soluble fraction (HS—HS-Tx—RIPA) relative to the insoluble fractions (2%SDS—Ac.Form) after SF normalization [means ± standard error of the mean (SEM)]

To gain insights into changes of FUS solubility, biochemical analysis was performed on frozen brain extracts of patient AH-11-02 and in two control individuals devoid of neurodegenerative disease. Proteins were extracted sequentially, using buffers with increasing solubilisation properties, as described in [57]. As expected, FUS could be detected as a 70-kDa band, and was mainly recovered in the most soluble fractions, as well as in the 2% SDS fraction. In the patient extracts, we noted a shift of FUS staining towards the more insoluble fractions, which resulted in a clear decrease of the soluble/insoluble ratio in the patient compared to controls (Fig. 3a and b).

**Fig.4:**
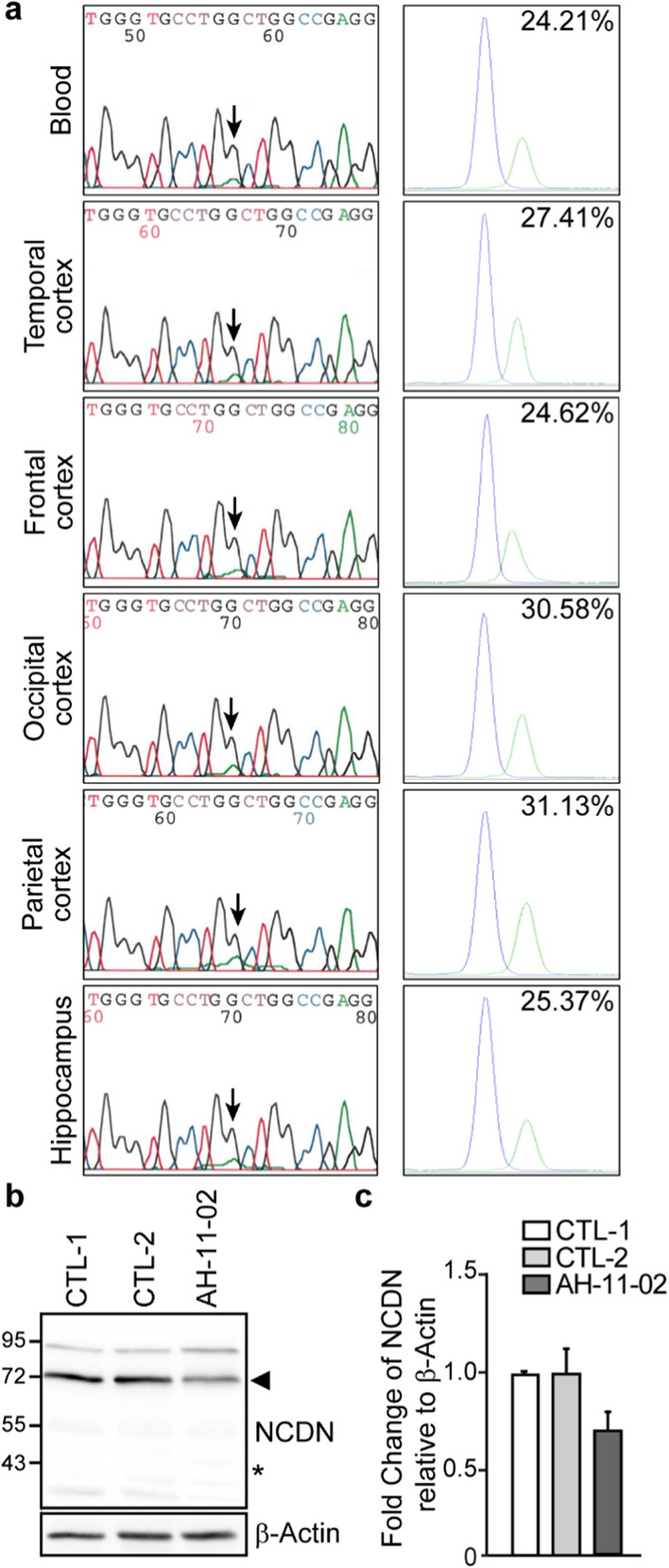
Detection of the *NCDN *de novo variant (c.1206G > A; p.(Trp402*)) in the FTLD-FET patient. **a** Confirmation of the variant in *NCDN* by Sanger sequencing (left panels) and SNaPshot (right panels) in blood and in different brain regions. In SNaPshot panels, blue represents the WT allele (G) and green the mutated allele (A). **b** Western blot analysis of NCDN and β-Actin from control (CTL-1, -2) or FTLD-FET affected patient (AH-11–02) frontal cortical tissues. Arrowhead indicates full-length NCDN and the star shows the absence of truncated NCDN at the expected size of 43KDa. **c** Quantification of NCDN expression shows a ~ 31% decrease in NCDN protein level from n = 4 experimental replicates per group. Error bars represent the mean ± SEM

To better understand the cause leading to FTLD-FET, we performed Sanger sequencing of the *FUS* gene in DNA isolated from blood and identified no candidate variant. In order to target de novo genetic variants responsible for causing FTLD-FET, we performed whole exome sequencing (WES) on DNA isolated from blood from the patient and her unaffected parents. We first explored all neurodegenerative dementia genes and found no putatively pathogenic variant in patient AH-11-02 (see methods). We then focused on candidate de novo variants. This led to the identification of a unique de novo variant, in the *NCDN* gene. This novel variant (NM_014284.3:c.1206G > A, p.(Trp402*); Chr1(GRCh37):g.36028055G > A) was indeed absent in the parental samples and present in 24.5% (12/49) of the reads in the proband, suggesting that the mutation could be post-zygotic in ~ 39% of the patient’s cells. We confirmed the presence of the variant by Sanger sequencing and using the SNaPshot technique in DNA extracted from blood and from different regions of the brain cortex of the patient (Fig. 4a), confirming that the mutation is an early post-zygotic event with an allelic ratio ranging from 24.6% to 31.1% in different brain cortical regions and 24.2% in blood.

This variant introduces a premature stop codon in exon 4 out of 7 exons regarding this transcript and is hence predicted to trigger nonsense-mediated decay (NMD). It is absent from the genome aggregation database (gnomAD, v2.1), gathering exomes and whole genomes of 141,456 individuals of diverse ethnicities [38]. More importantly, the *NCDN* gene appears to be very intolerant to loss-of-function variants, with an observed/expected ratio of 0.04 (upper boundary of the 95% confidence interval: 0.17) and a maximum loss of function intolerance score of 1 in gnomAD [38]. This indicates that the general population is highly depleted in truncating variants (nonsense, frameshift), as also further confirmed by the absence of any deletion in copy number variant databases including the Database of Genomic Variants (DGV, [50]) and gnomAD-SV [9]. When looking further into gnomAD, we could identify two frameshift variants seen 4 times each and a splicing variant observed once. Both frameshift variants mapped to the penultimate exon (exon 6/7) within the last 50 base pairs and were hence not predicted to trigger NMD. The splice site variant was observed once and mapped to the 5’ canonical splicing site of intron 6; it was also not predicted to trigger NMD if altered splicing would introduce a premature stop codon. Overall, this suggests that there is no human being reported in a control database so far with a truncating variant predicted to trigger NMD as is the case for the c.1206 > A, p.(Trp402*) variant.

We hypothesized that this de novo variant would result in haploinsufficiency. The *NCDN* gene encodes the 75 kDa Neurochondrin protein, also known as Norbin. *NCDN* is highly expressed in the brain and even appears to be brain-specific according to the Genotype-Tissue Expression (GTEX) database [10]. We assessed the expression of NCDN in the patient’s brain samples and found a ~ 31% reduction in full-length protein levels compared to control brains (Fig. 4b and c), consistent with our hypothesis.

In a replication attempt, we searched for *NCDN* variants in 25 additional unrelated unsolved FTLD-FET patients (see methods, Additional file 1: table 1) by whole exome or Sanger sequencing and identified no non-synonymous *NCDN* variant, suggesting that such variants remain extremely rare.

Overall, our analysis on this FTLD-FET patient led to the identification of a new rare *NCDN* variant associated with decreased NCDN protein levels in the brain, FUS inclusions and altered FUS solubility.

### NCDN haploinsufficiency affects FUS expression and localization in neurons

NCDN is highly expressed and distributed throughout the cytoplasm, axons, dendrites, spines and perisynapse and has roles in dendrite morphogenesis, neural outgrowth and synaptic plasticity [34, 61, 71, 72, 83, 84]. It has been shown to modulate metabotropic glutamate receptor 5 (mGluR5), through positively regulating mGluR5 cell surface expression and downstream signaling [84]. Importantly, FUS has also shown to be regulated through mGLUR1/5 activity and its downstream signaling pathways [19, 68, 69]. Upon, mGluR1/5 stimulation, FUS expression increases in dendrites and synapses of neurons [2, 19, 68]. Acting downstream of mGluR1/5 is the mechanistic target of rapamycin (mTOR) signaling pathway, which is the major regulator of cytoplasmic and local translation at synapses [28]. Under conditions where the mTOR pathway is inhibited, FUS is shown to localize to the cytoplasm where it promotes translation repression [69].

**Fig. 5:**
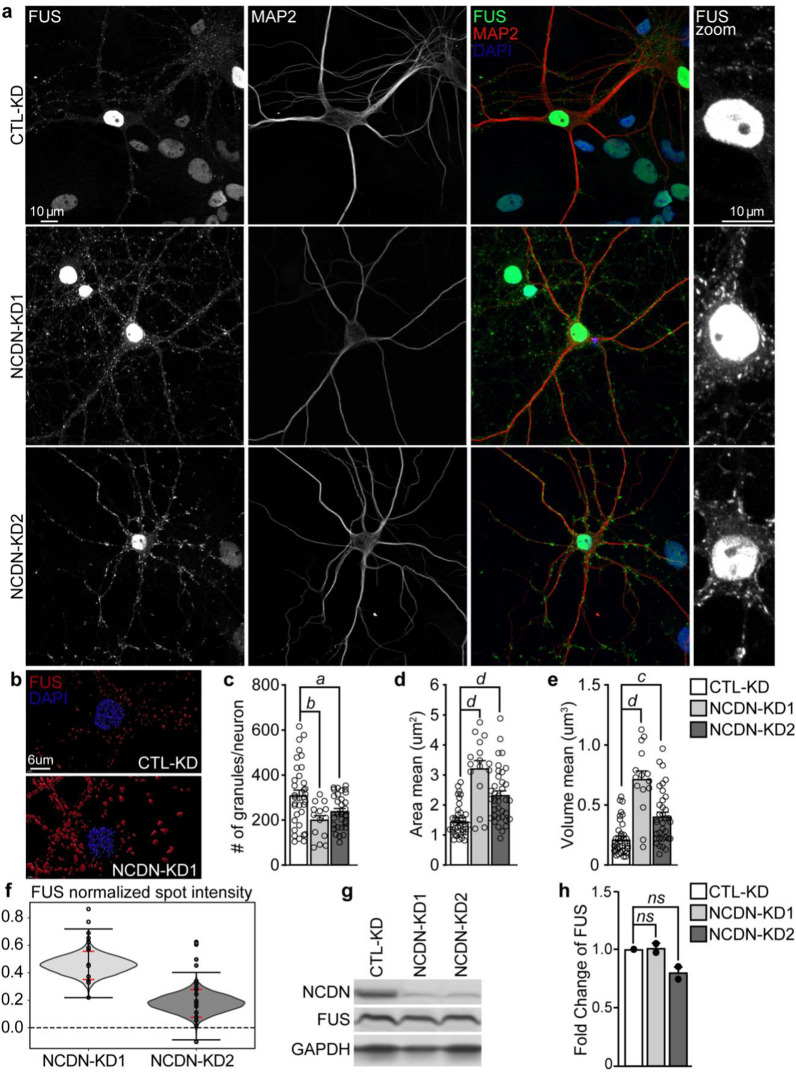
NCDN depletion in neurons affects FUS cytoplasmic granule dynamics. Lentivirus containing shRNAs targeting *NCDN* (NCDN-KD1 or -KD2) or non-targeted scramble (CTL-KD) were used to deplete primary rat cortical neurons (RCN) of *NCDN*. **a** Confocal images of RCN (DIV16) stained with antibodies against FUS (HPA008784, green), MAP2 (red) and DAPI (blue). Scale bar = 10 μm. **b** IMARIS generated 3D surface images of FUS-positive cytoplasmic granules from RCN. Quantification of the number of granules per neuron (**c**), mean area (**d**) and volume of granules (**e**). **f** Bootstrapped difference of maximum intensity of segmented FUS cytoplasmic granules from NCDN-KD compared to CTL-KD RCN showing the kernel density plot of the bootstrapped differences (shaded grey area), the minimum and maximum resampled differences (black horizontal lines), and the 95% confidence interval (red horizontal lines). NCDN-KD1,95% CI: 0.0062- 0.1937 *p* = 0.0098; NCDN-KD2,95% CI: 0.0044–0.1372 *p* < 0.0001. **g** Western blot of NCDN, FUS and GAPDH proteins from RCN. **h** Quantification of FUS protein levels from RCN relative to GAPDH. Statistical analysis was performed using a one-way ANOVA with multiple comparisons using Turkey test (**c**–**e**) or a Student’s t test (**h**) (*a*, *p* < 0.05; *b*, *p* < 0.01; *c*, *p* < 0.005; *d*, *p* < 0.001 vs CTL; *ns*, not significant, *p* > 0.05 vs CTL). Error bars represent the mean ± SEM. Each experiment was performed from n = 3–4 biological replicates per group

To determine the impact of NCDN haploinsufficiency on FUS pathology, we depleted primary rat cortical neurons of NCDN using lentiviruses containing shRNAs targeted against *NCDN* (NCDN-KD) and examined the effects on FUS localization and expression. We found that depleting neurons of NCDN resulted in changes in FUS-positive cytoplasmic granules (Fig. 5a and Additional file 1: Fig. S1 and S2). Analysis of these granules in NCDN-deficient neurons showed a significant reduction in the number and an increase in size of the FUS-positive granules when compared with control neurons (Fig. 5b–e). Moreover, the signal intensity of FUS within these structures was significantly increased (Fig. 5f), without any significant changes to the protein expression of FUS (Fig. 5g and h). These findings suggest that depleting neurons of NCDN affects FUS localization and association with cytoplasmic granules.

**Fig. 6:**
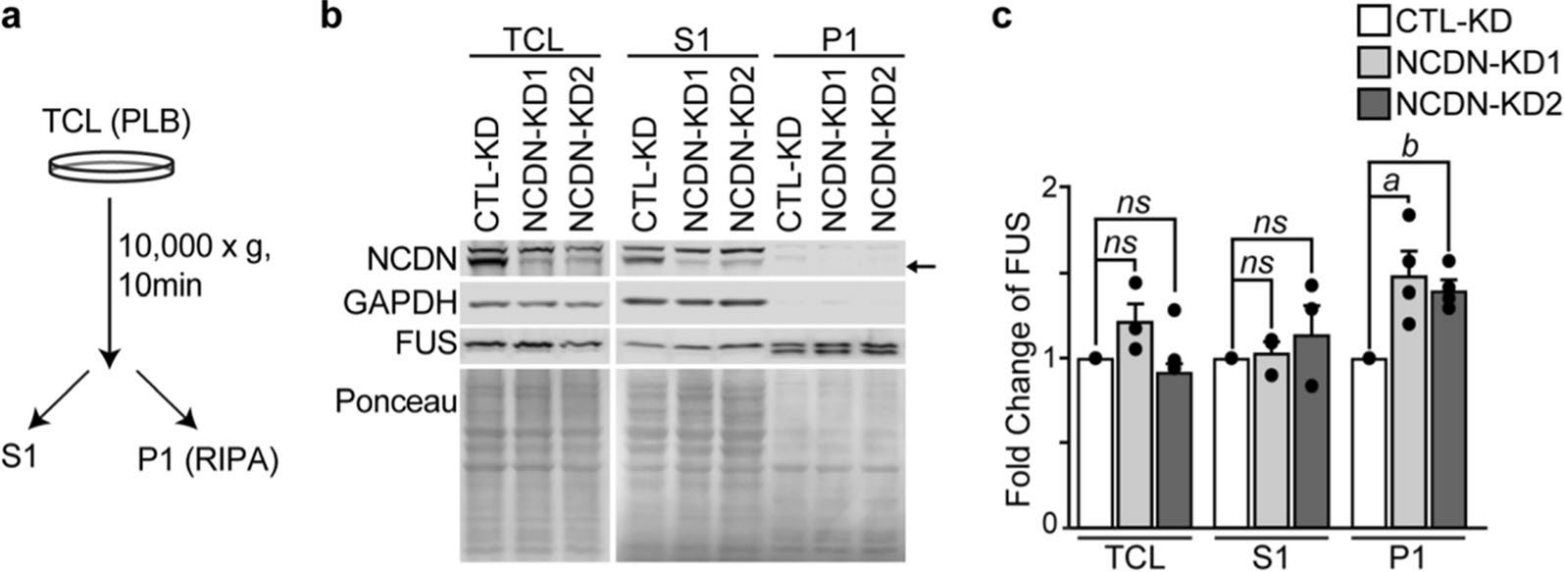
Loss of NCDN affects FUS localization and solubility**.** Lentivirus containing shRNAs targeting *NCDN* (NCDN-KD1 or -KD2) or non-targeted scramble (CTL-KD) were used to deplete Neuro-2a cells of *NCDN*. **a** CTL-KD and NCDN-KD cells were lysed in PLB followed by fractionation to generate soluble (S1) and insoluble fractions (P1). TCL: total cell lysates. PLB: polyribosome lysis buffer. RIPA: radioimmunoprecipitation assay buffer. **b** Western blot of NCDN, FUS and GAPDH proteins and corresponding ponceau red staining of membranes. 5% of each fraction was loaded on the gel. The arrow indicates the band corresponding to NCDN. **c** Quantification of FUS protein levels in fractions. Statistical analysis was performed using a Student’s t test (*a*, *p* < 0.05; *b*, *p* < 0.01; *ns*, not significant, *p* > 0.05 vs CTL). Error bars represent the mean ± SEM. Each experiment was performed from n = 3–4 biological replicates per group

To determine if the localization and solubility of FUS is affected by a reduction in NCDN, we isolated the soluble (S1) and insoluble (P1) fractions from NCDN-depleted Neuro-2a cells by subcellular fractionation (Fig. 6a). We examined the distribution of FUS in these fractions and found that FUS was enriched in the P1 fraction, of these cells (Fig. 6b and c), similar to what was observed in the patient (Fig. 3). Together, these data suggest that loss of NCDN can affect FUS localization and granule dynamics in cells.

### FUS loss-of-function affects NCDN expression in neurons

**Fig. 7:**
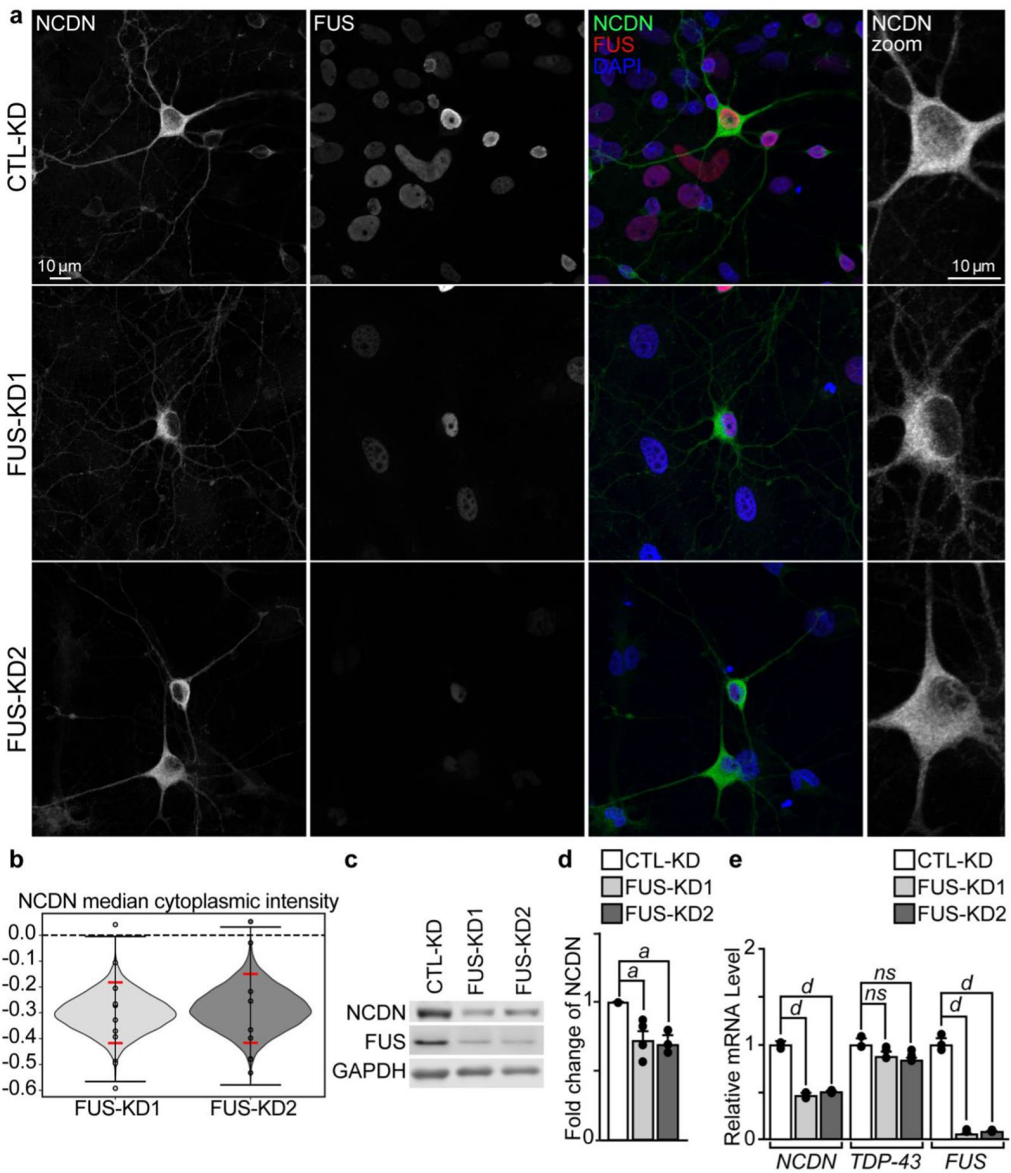
FUS depletion in neurons affects NCDN protein and mRNA levels. Lentivirus containing shRNAs towards FUS (FUS-KD1 or KD2) or non-targeted scramble (CTL) were used to infect primary rat neurons (RCN). **a** Confocal images of RCN (DIV16) stained with antibodies against FUS (sc-47711, red), NCDN (green) and DAPI (blue). Scale bar = 10 μm. **b** Bootstrapped difference of NCDN cytoplasmic intensity of medians from FUS-KD compared to CTL-KD RCN showing the kernel density plot of the bootstrapped differences (shaded grey area), the minimum and maximum resampled differences (black horizontal lines), and the 95% confidence interval (red horizontal lines) in neurons. FUS-KD1,95%CI: 0.0050- 0.1525 *p* = 0.0003; FUS-KD2,95% CI: 0.0057–0.1629 *p* = 0.001. **c** Western blot of NCDN, FUS and GAPDH proteins from RCN. **d** Quantification of NCDN protein levels from RCN relative to GAPDH. **e** Quantitative RT-PCR for *NCDN*, *TDP-43* and *FUS* relative to *U36B* from primary mouse cortical neurons (DIV16). Statistical analysis was performed using a Student’s t test (*a*, *p < *0.05; *d*, *p* < 0.001 vs CTL; *ns*, not significant, *p* > 0.05 vs CTL). Error bars represent the mean ± SEM. Each experiment was performed from n = 3–4 biological replicates per group

A pathological hallmark of FTLD-FET as well as ALS-FUS is the nuclear loss and cytoplasmic aggregation of FUS, which is thought to contribute to the disease due to a toxic loss of nuclear function and a toxic gain-of-cytoplasmic function [33, 48, 65, 68, 70]. As an approach to mimic the loss of FUS function shown to contribute to disease, we depleted cells of FUS and examined the effect on NCDN expression. We found that knocking-down FUS with lentiviruses containing FUS shRNAs (FUS-KD) did not affect the distribution of NCDN in primary rat cortical neurons (Fig. 7a), but significantly decreased the median fluorescence intensity of cytosolic NCDN in FUS-depleted neurons (Fig. 7a and b). These findings corresponded with global decreases in NCDN protein expression in these neurons (Fig. 7c and d).

FUS is shown to stabilize the expression of mRNAs [64, 79, 90] and dysregulation of FUS can lead to destabilization and NMD of its mRNA targets [25, 36, 92]. Therefore, we examined the mRNA expression of NCDN in FUS-depleted neurons and found there to be a reduction in total NCDN mRNA compared to control neurons (Fig. 7e), a finding that was also consistent in Neuro-2a cells (Additional file 1: Fig. S3). Consistent with previous reports, we did not observe any changes in TDP-43 mRNA levels in FUS-depleted neurons [39, 46, 65]. Together, these findings suggest that FUS is involved in the regulation of NCDN protein expression and/or mRNA stability.

## Discussion

We report here a de novo* NCDN* mutation in a sporadic FTLD-FET patient. This nonsense mutation was associated with reduced protein levels in the patient’s brain, hence haploinsufficiency as a main mechanism of disease. We thus investigated the relationship between NCDN and FUS and determined that a decrease in NCDN expression causes changes in FUS cytoplasmic granule dynamics. Moreover, our findings reveal that a decrease in FUS expression promotes a reduction in NCDN levels. Collectively, our data provide evidence for a negative feed-back loop of toxicity between NCDN and FUS, where loss of NCDN alters FUS cytoplasmic dynamics, and that the misregulation of FUS localization further promotes misregulation of NCDN expression (Additional file 1: Fig. S4). We conclude that FUS is downstream of NCDN. Exploring this pathway in the context of FTLD-FET is relevant to further our understanding of the disease and for the development of therapeutic targets.

The majority of familial FTD (~ 60%) cases are caused by autosomal dominant mutations in *GRN*, *MAPT* and *C9orf72* [21, 22]. The genetic cause of FTLD-FET is less clear and *FUS* mutations are exceptionally found in FTD patients [31, 66, 81]. To our knowledge, the *NCDN* mutation c.1206G > A, p.(Trp402*) identified in this study has not been previously reported in humans. Moreover, we could not identify any other case carrying a non-synonymous *NCDN* variant despite gathering 25 FTLD/ALS-FUS cases through international collaboration. FTLD-FET is among the rarest subtypes of FTLD and can itself be subdivided into different subgroups. Although our case was diagnosed with aFTLD-U, we conservatively explored other subtypes of FTLD-FET, namely NIFID and BIBD cases, along with aFTLD-U cases. However, it remains unclear whether NIFID, BIBD and aFTLD-U are closely related disorders that could share strong genetic determinants or if the molecular bases are distinct. Hence, the replication step of our analysis can be considered as limited, if *NCDN* truncating variants may be exclusively associated to aFTLD-U.

FTD shares clinical, genetic and pathogenic features with ALS [37, 52]. ALS is caused by degeneration of upper and lower motor neurons leading to progressive paralysis and death [3]. Unlike FTLD-FET, autosomal dominant mutations in *FUS* account for 5–10% ALS [15, 44, 82]. Most ALS-FUS mutations result in an increase in cytoplasmic FUS and the formation of FUS-positive cytoplasmic inclusions [15, 44, 82], similar to those found in FTLD-FET [31, 51, 66]. Our identification of this de novo mutation in *NCDN* suggests that a pathway related to NCDN could contribute to FUS mislocalization, which may partly explain how patients can display FUS-associated degenerative defects without having mutations in the *FUS* gene. A number of de novo mutations in sporadic ALS patients have been identified in ALS trios studies, although none affected the *NCDN* gene, to our knowledge [8, 73, 80]. In the ALS variant server, which gathers genetic data of 1277 ALS cases, three missense *NCDN* ultra rare variants are reported (NM_014284.3:c.1417G > A, p.(Val473Ile) in a sporadic ALS patient, c.509G > A, p.(Arg170Gln) in a familial ALS patient and c.1507C > G, p.(Pro503Ala) in 3 familial ALS patients), but their inheritance is not mentioned and they are mainly predicted as benign by bioinformatics tools. No truncating variant is reported on this server (ALS Variant Server, Worcester, MA (URL: http://als.umassmed.edu/) [nov, 2021 accessed].

Until very recently, *NCDN* was not associated with any Mendelian disorder. Missense variants were however reported in patients with a neurodevelopmental phenotype with epilepsy. One family exhibited a missense homozygous variant, with unaffected heterozygous parents, while 3 unrelated patients showed missense de novo variants with a more severe phenotype [18]. Functional analyses suggested a loss-of-function effect, the bi-allelic variant being most likely hypomorphic, and de novo variants affected NCDN function more severely. The authors suggested that two of the variants, located in the mGluR5 binding domain, specifically altered this pathway. It remains unclear whether haploinsufficiency is the most accurate model to mimic these missense variants’ effects, although it is clear that they are pathogenic through loss of *NCDN* function. In addition, although there are no truncating *NCDN* variant predicted to trigger NMD in variant databases or deletions in controls, a few patients with a developmental disorder were reported with deletions encompassing *NCDN* and multiple other genes. However, it is unclear (i) whether *NCDN* plays a role in the developmental phenotype of the latter patients and (ii) whether the same patients as well as recently described patients with missense variants will eventually develop FTLD-FET. Here, the nonsense mutation was detected as a mosaic. We hypothesize that such an NMD-triggering variant, if carried in the germ line, might be lethal during in-utero development, which could explain absence from control databases and absence of association with developmental disorders.

In a non-Mendelian manner, NCDN has been implicated in several neurological disorders including epilepsy, depression, schizophrenia and spinal muscular atrophy (SMA) [1, 12, 53, 59, 76, 83–85, 87]. Previous studies in mice have shown that forebrain-specific knockout of NCDN causes impaired mGluR5 dependent long-term potentiation (LTP) and long-term depression (LTD), as well as schizophrenia-like phenotypic behaviors [84]. Neuron-specific NCDN knockout mice have reduced hippocampal neurogenesis and depressive-like behaviours [83]. Overexpression of NCDN in neuronal cells induces neurite outgrowth [72]. Collectively, these studies show that NCDN has important roles in dendrite morphogenesis, neural outgrowth and synaptic plasticity [34, 61, 71, 72, 83, 84]. Our data show that depleting NCDN from neurons affects FUS cellular distribution and association with cytoplasmic granules. While the changes in localization of FUS in our neuron cultures did not cause cell death, mislocalization of FUS to the cytoplasmic compartment has been shown to cause loss of dendritic branching, loss of mature spines and contribute to neurodegeneration [32, 48, 65, 68, 70]. Taken together, these findings support the hypothesis that NCDN and FUS are part of the same regulator pathway important for neuronal function.

NCDN is also linked with SMA, a degenerative muscular disease caused by a reduction in the survival motor neuron (SMN) full-length protein [47, 76]. The findings from one study show that the function and expression of NCDN and SMN are co-dependent [76]. When SMN levels are reduced in cells, it causes a decrease in the amount of cytoplasmic NCDN-positive granules. Conversely, depleting cells of NCDN leads to an increase in SMN nuclear foci in SH-SY5Y cells. Moreover, this study identified that NCDN and SMN interact within mobile cytoplasmic granules [76]. Interestingly, FUS and SMN are shown to share a common pathway in the context of neurobiology and ALS [6, 7, 23, 88]. FUS is found to associate with the SMN complex through a direct interaction with SMN [88]. Functionally, overexpression of SMN can rescue the neurite growth defects and reduced dendritic branching induced by ALS-FUS mutants [7]. In our study we show that depleting cells of FUS affects NCDN protein and mRNA levels (Fig. 7). Our findings are consistent with published supplemental data from the brains of FUS KO mice that shows a decrease in NCDN mRNA [65]. Moreover, NCDN mRNA is among the list of genes found to interact with FUS [46], an interaction that remains to be validated. Further analysis is required to determine if FUS and SMN are directly regulating *NCDN* translation and whether NCDN:FUS:SMN are part of a converging pathway involved in maintaining synaptic homeostasis.

A previous study showed that NCDN interacts with a subset of group 1 mGluRs, including mGluR5, where NCDN has been shown to promote mGluR5 cell surface expression and activation of mGluR5 through a direct interaction with this receptor [84]. mGluR5 activation at spines has been shown to regulates local protein synthesis at the synapse, which promotes synaptic strength and promote LTD [30, 86]. Ligand activation of mGluR5 initiates several downstream signaling pathways including the PI3K/AKT/mTOR pathway [28, 29]. In neurons, the PI3K/AKT/mTOR pathway is the central pathway for regulating local translation and synaptic strength [24, 26]. The activity of FUS is shown to be modulated by mGluR1/5 [19, 68, 77]. When neurons are treated with an mGluR1/5 agonist, FUS localization and expression increases locally at spines [19, 68], which does not occur in *mGluR5*^−/−^ neurons [19]. In response to glutamate excitotoxicity, where mGluRs are overactivated, FUS localizes to the cytoplasm of neurons, which correlates with a global decrease in protein synthesis [77]. Under conditions where the mTOR pathway is pharmacologically inhibited, FUS is shown to localize to the cytoplasm where it associates with stalled polyribosomes and promotes translation inhibition [69]. In our study we find that under conditions where NCDN is depleted in neurons, the localization and cytoplasmic dynamics of FUS is affected. Moreover, our findings in cell culture are consistent with the presence of cytoplasmic FUS inclusions in a FTLD-FET patient with NCDN haploinsufficiency. Our findings suggest that similar to mGluR1/5 activation, which affects FUS cytoplasmic distribution and expression [19, 68, 77], NCDN expression also affects FUS localization and activity, which might be due to the lack of signaling of mGluR5 at the cell surface (Additional file 1: Fig. S4) [84].

## Conclusions

In summary, we have identified a rare de novo* NCDN* variant in a FTLD-FET patient that results in haploinsufficiency of NCDN. We conclude that NCDN has an important function in regulating FUS granule dynamics, and that these changes cause a misregulation of NCDN expression. Based on the common biological functions of both NCDN and FUS, as well as their link with FTLD-FUS and ALS-FUS, there is an intriguing possibility that these proteins are part of a common regulatory pathway for the maintenance of synaptic homeostasis. Taken together, our findings suggest that disruption of this pathway would lead to neuronal defects and neurodegeneration.

## Supplementary Information

**Additional File 1: Supplementary tables and figures:** **Table S1**. Exome sequencing data or DNA samples from multiple international cases with FTLD-FET. **Fig. S1**. Validation of secondary antibody specificity for immunocytochemistry studies. **Fig. S2.** Immunocytochemistry validation of NCDN knock-down in neurons. **Fig. S3**. Knock-down of FUS in N2a affects NCDN protein and mRNA levels. **Fig. S4**. Model for NCDN haploinsufficiency and FTD-FET. **Supplementary References**. Citations for Table S1.

## Data Availability

The microscopy image datasets supporting the conclusions of this article are available at https://s3.valeria.science/ncdn-fus/index.html. All codes required to perform quantitative image analysis are available at https://github.com/FLClab/NCDN-FUS. All other data generated or analysed during this study are included in this published article.

## Abbreviations

FTD: Frontotemporal dementia
FTLD: Frontotemporal lobar degeneration
TDP: TAR-DNA binding protein-43
FUS: Fused in sarcoma
EWS: Ewing Sarcoma Breakpoint region 1
TAF15: TATA-binding protein-associated factor 15)
aFTLD-U: Atypical-FTLD with ubiquitin-positive inclusions
BIBD: Basophilic inclusion body disease
NIFID: Neuronal intermediate filament inclusion body disease
ALS: Amyotrophic lateral sclerosis
PY-NLS: Proline-tyrosine nuclear localization sequence
NES: Nuclear export sequence
RRM: RNA recognition motif
ZnF: Zinf finger
RRG: Arginine-glycine-glycine
LCD: Low complexity domain
LLPS: Liquid–liquid phase separation (LLPS)
mGluR1/5: Metabotropic glutamate receptor group 1 subtypes 1 and 5
mTOR: Mechanistic target of rapamycin
NCDN: Neurochondrin
WES: Whole exome sequencing
NMD: Nonsense-mediated decay
DGV: Database of genomic variants
GTEX: Genotype-tissue expression
SMA: Spinal muscular atrophy
LTP: Long-term potentiation
LTD: Long-term depression
SMN: Survival motor neuron
