# Supplementary material for: A postzygotic de novo NCDN mutation identified in a sporadic FTLD patient results in neurochondrin haploinsufficiency and altered FUS granule dynamics
Source: Acta Neuropathol Commun. 2022 Feb 12;10:20. doi: 10.1186/s40478-022-01314-x (PMC8841087; doi:10.1186/s40478-022-01314-x)
Supplement: Supplementary file 1 — Additional File 1: Supplementary tables and figures: Table S1. Exome sequencing data or DNA samples from multiple international cases with FTLD-FET. Fig. S1. Validation of secondary antibody specificity for immunocytochemistry studies. Fig. S2. Immunocytochemistry validation of NCDN knock-down in neurons. Fig. S3. Knock-down of FUS in N2a affects NCDN protein and mRNA levels. Fig. S4. Model for NCDN haploinsufficiency and FTD-FET. Supplementary References. Citations for Table S1. [file 40478_2022_1314_MOESM1_ESM.pdf]

SUPPLEMENTARY TABLES AND FIGURES

Table S1. Replication analysis: clinical information. bvFTD: behavioral variant of fronto-temporal dementia, NIFID: Neuronal intermediate filament inclusion disease, aFTLD-U: atypical Fronto-temporal degeneration with ubiquitin-positive inclusions, BIBD: basophilic inclusion body disease, FTLD-FET: Fronto-Temporal Lobar Degeneration, FET-type (uncharacterized), WES: whole exome sequencing, PSP: progressive supranuclear palsy, ALS: amyotrophic lateral sclerosis.

Patient	Country	Patient code	Sequencing method	Tissue for sequencing	Sex	Age of onset	Age of death	Pathological diagnosis	Clinical presentation
2	France / Lille	EXT 353	WES	Blood	F	32	35	NIFID	bvFTD
3	France / PSL	FUS 007	WES	Brain	M	42	45	NIFID	Subacute bvFTD
4	France / PSL	FUS 008	WES	Brain	M	33	35	NIFID	bvFTD with motor and extrapyramidal deficits
5	France / PSL	FUS 009	WES	Brain	M	57	59	NIFID	Motor deficit and psychiatric symptoms
6	France / Rouen	ROU 369	WES	Blood	F	53	64	FTLD-FET	bvFTD
7	France / PSL	FUS 4954	WES	Brain	F	NA	NA	FTLD-FET	Dementia
8	Spain / Barcelona	FUS-10402070[3]	WES	Brain	M	64	64	NIFID	bvFTD
9	Spain / Barcelona	FUS-10800070[2]	WES	Brain	M	76	81	NIFID	bvFTD + PSP
10	Spain / Barcelona	FUS-300070[3]	WES	Brain	M	37	44	NIFID	bvFTD
11	Spain / Barcelona	FUS-508000[3]	WES	Brain	M	38	43	BIBD	bvFTD
12	Spain / Barcelona	FUS-700020[3]	WES	Brain	F	70	75	NIFID	Atypical parkinsonism
13	Spain / Barcelona	FUS-1040105[1]	Sanger	Brain	M	43	48	BIBD	ALS
14	Spain / Barcelona	FUS-1060809[1]	Sanger	Brain	M	63	69	BIBD	ALS
15	Spain / Barcelona	FUS-1070500[1]	Sanger	Brain	F	71	83	BIBD	ALS
16	Japan	Case 8[4]	Sanger	Brain	M	29	37	NIFID	bvFTD
17	Japan	Case 1[4]	Sanger	Brain	F	32	39	BIBD	bvFTD
18	France / Lille	FUS 003	Sanger	Brain	M	40	44	aFTLD-U	bvFTD
19	France / Lille	FUS 004	Sanger	Brain	M	48	54	aFTLD-U	bvFTD
20	France / Lille	FUS 002	Sanger	Brain	F	50	55	aFTLD-U	bvFTD
21	France / Lille	FUS 001	Sanger	Brain	F	31	34	aFTLD-U	bvFTD
22	Netherlands	NL_P1	WES	Blood	F	35	45	aFTLD-U	bvFTD
23	Netherlands	NL_P2	WES	Blood	F	36	38	aFTLD-U	bvFTD
24	Netherlands	NL_P3	WES	Blood	F	32	40	aFTLD-U	bvFTD
25	Netherlands	NL_P4	WES	Blood	F	30	45	aFTLD-U	bvFTD
26	Netherlands	NL_P5	WES	Blood	M	43	49	aFTLD-U	bvFTD

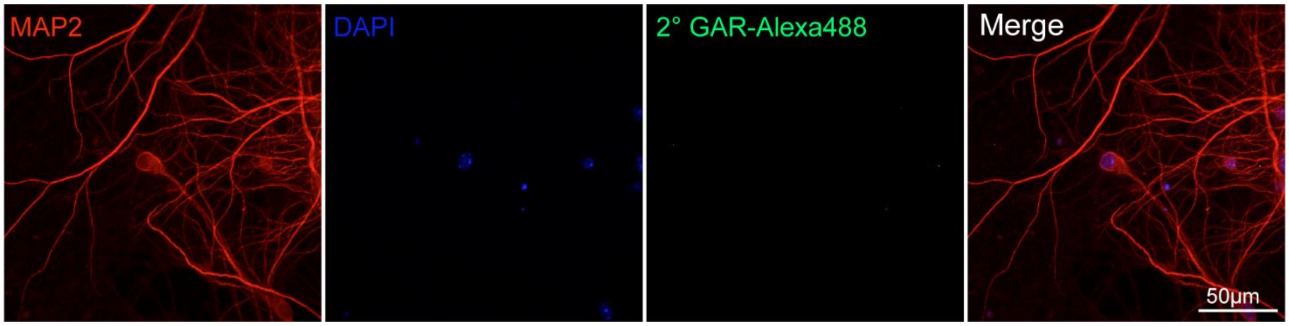

Fig. S1 Validation of antibody specificity for the secondary rabbit polyclonal antibody used to detect FUS. Confocal images of primary rat cortical neurons (DIV 16) stained with MAP2 (red), DAPI (blue) and the goat anti-rabbit (GAR) secondary antibody alone (2° GAR-Alexa488, green). Scale bar = 50 μm.

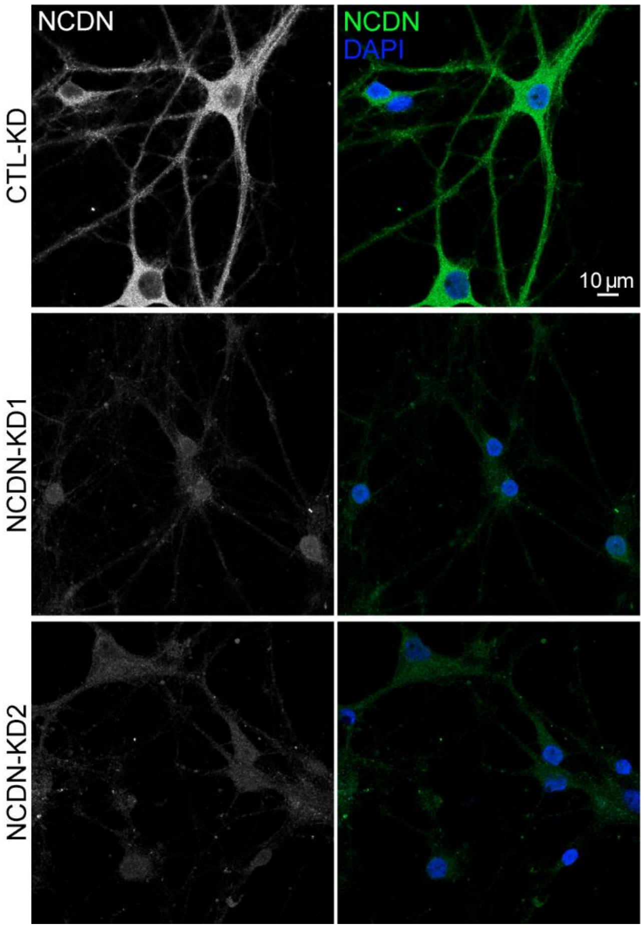

Fig. S2 NCDN knock-down in neurons. Lentivirus containing shRNAs targeting *NCDN* (NCDN-KD1 or -KD2) or non-targeted scramble (CTL-KD) were used to infect primary rat cortical neurons (RCN). Confocal images of RCN (DIV16) stained with antibodies against NCDN (green) and DAPI (blue). Scale bar = 10 µm.

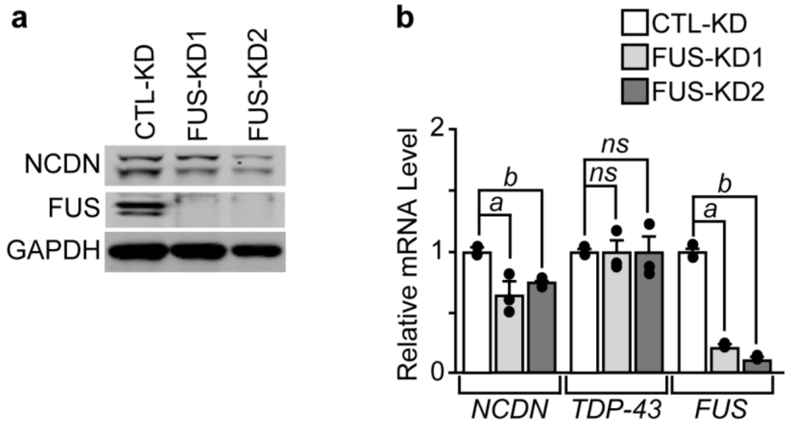

Fig. S3 FUS depletion in Neuro-2a cells affects NCDN protein and mRNA levels. Lentivirus containing shRNAs towards FUS (FUS-KD1 or KD2) or non-targeted scramble (CTL) were used to infect cells. **a** Western blot of NCDN, FUS and GAPDH proteins from Neuro-2a cells. **b** Quantitative RT-PCR for *NCDN*, *TDP-43* and *FUS* relative to *U36B* from Neuro-2a cells. Statistical analysis was performed using a Student's t test (*a*, $p<0.05$; *d*, $p<0.001$ vs CTL; *ns*, not significant, $p>0.05$ vs CTL). Error bars represent the mean ± SEM. Each experiment was performed from n=3-4 biological replicates per group.

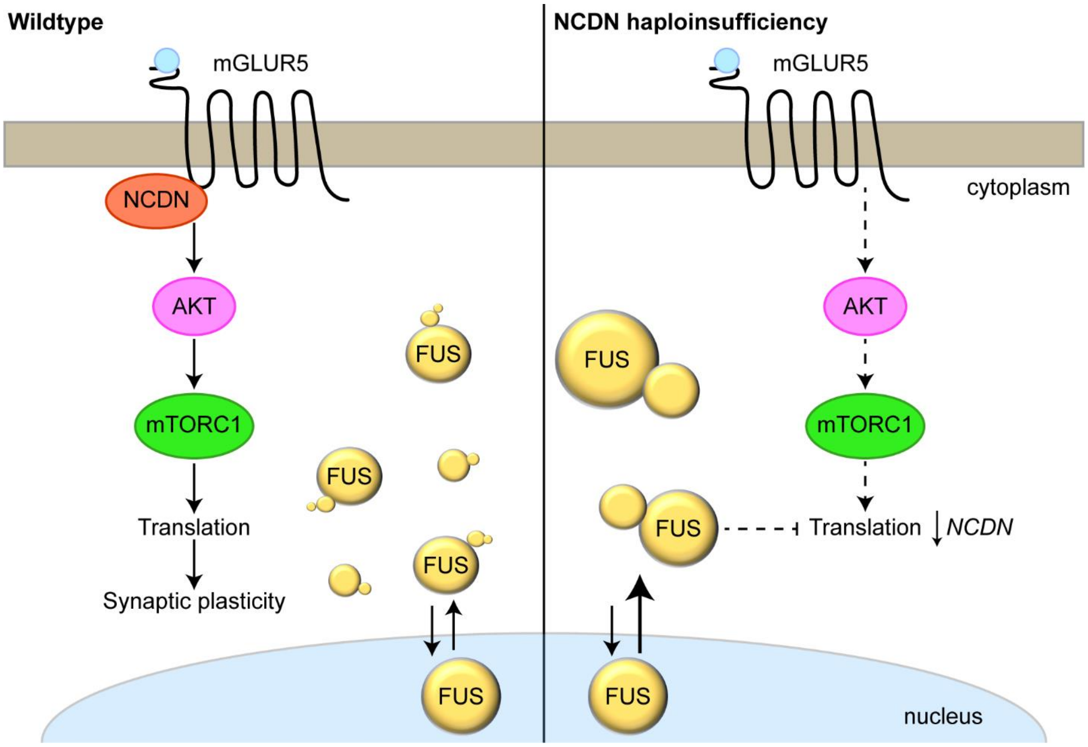

Fig. S4 Model for NCDN haploinsufficiency and FTD-FET. In the wildtype condition (left panel), NCDN interacts with mGluR5 and is responsible for mGluR5 surface localization and mGluR5 signaling. Shown is mGluR5 signaling through the PI3K/AKT/mTORC1 signaling pathway, which in neurons is the central pathway for regulating translation and synaptic plasticity. Under these conditions FUS associates with cytoplasmic granules and undergoes nucleocytoplasmic trafficking in response to cellular cues. The *NCDN de novo* variant (p.Trp402*) causes NCDN haploinsufficiency (right panel), which results in less mGluR5 surface localization and defective signal transduction through PI3K/AKT/mTORC1. Under these conditions, FUS associates with larger and fewer cytoplasmic granules, which corresponds with lower NCDN protein and mRNA levels. Our findings provide evidence for a negative feed-back loop of toxicity between NCDN:FUS, where loss of NCDN alters FUS cytoplasmic dynamics and FUS loss-of-function can further promote neuronal dysfunction through the misregulation of NCDN expression.

SUPPLEMENTARY REFERENCES

1 Borrego-Ecija S, Cortes-Vicente E, Cervera-Carles L, Clarimon J, Gamez J, Batlle J, Ricken G, Molina-Porcel L, Aldecoa I, Sanchez-Valle Ret al (2019) Does ALS-FUS without FUS mutation represent ALS-FET? Report of three cases. Neuropathology and applied neurobiology 45: 421-426 Doi 10.1111/nan.12527

2 Compta Y, Ramos-Campoy O, Grau-Rivera O, Colom-Cadena M, Clarimon J, Marti MJ, Gelpi E (2017) Conjoint FTLD-FUS of the neuronal intermediate filament inclusion disease type, progressive supranuclear palsy and Alzheimer's pathology presenting as parkinsonism with early falls and late hallucinations, psychosis and dementia. Neuropathol Appl Neurobiol 43: 352-357 Doi 10.1111/nan.12340

3 Gelpi E, Llado A, Clarimon J, Rey MJ, Rivera RM, Ezquerra M, Antonell A, Navarro-Otano J, Ribalta T, Pinol-Ripoll Get al (2012) Phenotypic variability within the inclusion body spectrum of basophilic inclusion body disease and neuronal intermediate filament inclusion disease in frontotemporal lobar degenerations with FUS-positive inclusions. Journal of neuropathology and experimental neurology 71: 795-805 Doi 10.1097/NEN.0b013e318266efb1

4 Kawakami I, Kobayashi Z, Arai T, Yokota O, Nonaka T, Aoki N, Niizato K, Oshima K, Higashi S, Katsuse Oet al (2016) Chorea as a clinical feature of the basophilic inclusion body disease subtype of fused-in-sarcoma-associated frontotemporal lobar degeneration. Acta neuropathologica communications 4: 36 Doi 10.1186/s40478-016-0304-9
